# Co-Expression of HER Family Members in Patients with Dukes’ C and D Colon Cancer and Their Impacts on Patient Prognosis and Survival

**DOI:** 10.1371/journal.pone.0091139

**Published:** 2014-03-07

**Authors:** Said Abdullah Khelwatty, Sharadah Essapen, Izhar Bagwan, Margaret Green, Alan Michael Seddon, Helmout Modjtahedi

**Affiliations:** 1 School of Life Sciences, Kingston University London, Kingston, United Kingdom; 2 St Luke’s Cancer Centre, Royal Surrey County Hospital, Guildford, United Kingdom; 3 Department of Histopathology, Royal Surrey County Hospital, Guildford, United Kingdom; Sapporo Medical University, Japan

## Abstract

The human epidermal growth factor receptor (EGFR) is an important therapeutic target in patients with metastatic colorectal cancer and anti-EGFR antibodies cetuximab and panitumumab have been approved for the treatment of such patients. Despite these advances, the duration of response in some patients can be limited. Since, EGFR is capable of forming heterodimers with the other members of the HER (Human epidermal receptor) family, it is important to investigate the co-expression and prognostic significance of all members of the HER family in colorectal cancer patients. The expression of the HER family members were determined in tumour specimens from 86 patients with Dukes’ C and D (metastatic) colon cancer using immunohistochemistry. Sections were scored by the percentage of positive tumour cells and intensity of staining. Their associations with clinicopathological parameters, and overall survival and disease free survival were evaluated using univariate and multivariate analysis. Overall, 43%, 77%, 52% and 92% of the cases were EGFR, HER-2, HER-3 and HER-4 positive respectively. Interestingly, 35%, 24%, 43%, and 18% of the cases had co-expression of EGFR/HER-2, EGFR/HER-3, EGFR/HER-4 and all four members of the HER family respectively. Of these, only the expression of EGFR and co-expression of EGFR/HER-4 were associated with poorer disease-free survival in both univariate and multivariate analysis. Co-expression of all members of the HER family in colon cancer supports the need for further investigations on their predictive value for response to therapy with anti-EGFR mAbs and whether such sub-population of patients may benefit from therapy with the new generation of pan-HER inhibitors.

## Introduction

Colorectal cancer remains one of the leading causes of cancer deaths worldwide. In 2013, colorectal cancer is estimated to be the fourth most commonly diagnosed cancer (142,820) but the second leading cause of cancer deaths (50,830) after lung cancer in the USA [1]. Currently, of the various drugs developed for the targeted therapy of human cancers, the anti-epidermal growth factor receptor (EGFR) monoclonal antibodies (mAbs), cetuximab and panitumumab, and the anti-vascular endothelial growth factor (VEGF) mAb bevacizumab have been incorporated into treatment paradigms for the majority of patients with metastatic colorectal cancer [2]–[5]. While the inclusion of these agents has improved the survival of patients with metastatic colorectal cancer, the duration of response can be limited. In addition, there has been no reliable predictive marker for response to these anti-EGFR targeted therapies [6]–[10]. The development and identification of such markers not only could aid in the selection of a more specific sub-population of colorectal cancer patients who are more likely to benefit from such therapies, but they may also reduce unnecessary treatments and thereby the high cost to the healthcare system [11].

In the past four decades, the aberrant expression of different members of the HER family and their ligands have been reported in a variety of human cancers. In some studies, these have been associated with resistance to conventional forms of therapy and a poorer prognosis [6], [12], [13]. However, there is a wide variation in the reported expression of the HER family members in colorectal cancer patients [6], [14]–[16]. In addition to the formation of homodimers, the HER family members such as the EGFR are capable of being activated by forming heterodimers with other members of the HER family [17]–[19].

While a limited number of studies have investigated the expression and prognostic significance of individual members of the HER family in patients with colorectal cancer [20]–[23], however, to our knowledge, there have been no comprehensive studies on the co-expression and prognostic significance of the complete members of HER family in colorectal cancer patients to date [6]. Therefore, in this study we have investigated the expression levels of all members of the HER family, individually as well as their co-expression in tumour specimens from 86 patients with Dukes’ C and D colon cancer. We also investigated any association between the expression of the HER family members and the clinicopathological parameters, disease free survival and overall survival.

## Materials and Methods

### Patient Information

Ethical approval was obtained from the Research and Development Committee of the Royal Surrey County Hospital for examination of tumour specimens from patients with colon cancer for use in this retrospective study. As only archived tumour specimens were included in this study, the ethics committee waived the need for consent and patient records/information were analysed anonymously. Eighty-six patients with Dukes’ C and D colon cancer, who underwent radical surgery at the Royal Surrey County Hospital (Guildford, UK) between April 2002 and November 2007, were included in this retrospective study. Those with no follow-up information, mis-diagnosis, and incomplete histology were excluded. Cases of peri- and post-operative death were also excluded from this study, as were those with tumour blocks in a condition too poor for immunohistochemical use. Detailed clinicopathological information, including patient age and gender was available for each patient.

### Immunohistochemistry

Formalin fixed paraffin-embedded (FFPE) sections of tumour specimens (3 µM) were cut in serial sections and were stained using the following primary antibodies mouse anti-EGFR (1∶10, Novacastra, UK), mouse anti-HER-2 (1∶150, Insight biotechnology, UK), mouse anti-HER-3 (1∶20, Novacastra, UK) and rabbit anti-HER-4 (1∶20, Fisher Scientific, UK). Following antigen retrieval, tumour sections were incubated with primary antibodies anti EGFR, HER-3 and HER-4 for 60 minutes and HER-2 for 32 minutes. Protocol optimisation was carried using established HER positive cancer cell line pellets, namely the EGFR overexpressing human colorectal cancer cell line DiFi, which was kindly provided by Dr Z Fan (MD-Anderson Cancer Centre, USA), the HER-2 overexpressing human breast carcinoma cell line SKBR3 (HER-2), and the HER-3 and HER-4 positive human breast carcinoma cell line MCF-7 as described previously [24]. Staining was carried out on a Venatana Benchmark XT autostainer with the ultraView DAB kit (Roche, UK). Finally, all slides were rehydrated and counterstained with haematoxylin, mounted and cover slipped.

### Scoring System

In the current literature, the cut-off values for scoring positive HER immunostaining of tumour sections is variable. In this study, the immunostaining of the tumour sections were scored based on the percentage of tumour cells that had HER immunostaining (i.e. >5%, >10%, and >50%) and intensity of immunostaining (i.e. negative 0, weak positive 1+, moderately positive 2+ and strongly positive 3+) and whether the staining was predominantly present in the membrane, cytoplasm or nucleus of the cells [15]. Of the HER immunostaining, HER-4 had the highest levels of background staining and therefore immunostaining above the background level only was considered [22]. Two independent observers (including a consultant histopathologist), without prior knowledge of the clinicopathological parameters, conducted the scoring and any disparity in scoring was resolved by simultaneous reassessment of the staining by both observers.

### Statistical Analysis

The association between immunohistochemistry scores and patient clinicopathological data was assessed using Chi-Squared test (Pearson Chi-Square) and Fishers exact test. Kaplan-Meier survival plots were used to perform univariate analysis and the differences between groups was evaluated by performing log rank-test. For multivariate analysis, the Cox multi regression model was used and P≤0.05 was considered statistically significant. All statistical analyses were carried out using the PASW statistics 21 (SPPS Inc.).

## Results

### Clinicopathological Features

Patient clinicopathological characteristics are summarised in Table 1. The median patient follow-up time was 6 years. None of the patients had received radiotherapy or chemotherapy prior to surgery. Fifty-two patients received post-operative adjuvant chemotherapy, which was predominantly 5-fluorouracil based with some patients receiving oxaliplatin and irinotecan based therapies. A poorer overall survival was observed in patients with Dukes’ D compared with Dukes C (3.2±0.6 versus 6.2±0.4 years, *P = 0.005*), and those with more than 3 positive lymph nodes (4.3±0.4 versus 6.7±0.5 *P = 0.008*). No significant association was found between patient outcome and the other clinicopathological parameters (Table 1.).

**Table 1:** Clinicopathological parameters and survival of Dukes’ C and D colon cancer patients.

Characteristics	Number of patients(%)	Overall survivalin years (mean ± SE)	95% CI	*P-value*	Disease-free survival in months(mean ± SE)	95%CI	*P-value*
**Age in years**							
≤70	24 (28)	3.9±0.5	2.9–5.0	*NS*	66.8±6.6	53.7–79.8	*NS*
>70	62 (72)	6.0±0.6	5.2–6.9		85.1±5.4	74.4–95.9	
**Gender**							
Male	49 (57)	5.5±0.5	4.6–6.6	*NS*	84.9±6.5	72.2–97.6	*NS*
Female	37 (43)	5.8±0.6	4.7–7.0		80.0±7.5	65.3–94.8	
**Tumour Site**							
Right colon	44 (51)	5.3±0.5	4.2–6.4	*NS*	83.1±6.9	69.4–96.7	*NS*
Left colon	42 (49)	6.1±0.5	5.1–7.1		83.1±6.7	69.9–96.3	
**Dukes’ stage**							
C	67 (78)	6.2±0.4	5.4–7.0	*0.005*	82.3±5.5	71.6–93.0	*NS*
D	19 (22)	3.2±0.6	1.9–4.4		71.2±6.3	58.7–83.6	
**T stage**							
T4	22 (26)	4.1±0.7	2.8–5.5	*NS*	80.7±7.1	66.7–94.6	*NS*
<T4	64 (74)	6.1±0.4	5.3–6.9		80.7±5.7	69.4–91.9	
**M stage**							
M1	11 (13)	3.6±0.6	2.3–4.8	*NS*	62.5±7.1	48.6–76.6	*NS*
M0	75 (87)	5.9±0.4	5.1–6.7		83.6±5.3	73.3–93.9	
**Positive LN**							
>3	42 (49)	4.3±0.4	3.5–5.2	*0.008*	65.6±6.1	53.6–77.6	*NS*
≤3	44 (51)	6.7±0.5	5.7–7.7		93.3±5.7	82.1–104.5	
**LVI**							
Absent	58 (67)	6.1±0.4	5.3–7.0	*NS*	86.1±5.7	74.9–97.3	*NS*
Present	28 (33)	3.9±0.5	2.9–4.9		62.0±6.3	49.6–74.4	
**Grade**							
G3	50 (58)	5.2±0.5	4.2–6.2	*NS*	82.4±6.4	69.8–94.8	*NS*
<G3	36 (42)	6.3±0.6	5.2–7.4		81.9±7.9	66.4–97.3	
**Chemotherapy**							
No	23 (27)	4.1±0.5	3.0–5.2	*NS*	62.1±6.5	49.4–74.8	*NS*
Yes	52 (60)	6.2±0.5	5.3–7.1		85.1±6.1	73.2–97.1	
unknown	11 (13)	4.1±0.8	2.5–5.7		66.9±8.9	49.3–84.5	

Overall survival and disease-free survival relative to the indicated features was determined by Kaplan-Meier analysis and the log-rank test. P-value of *≤0.05* was considered significant.

### Immunohistochemical Expression of HER Family Members

**Figure 1:**
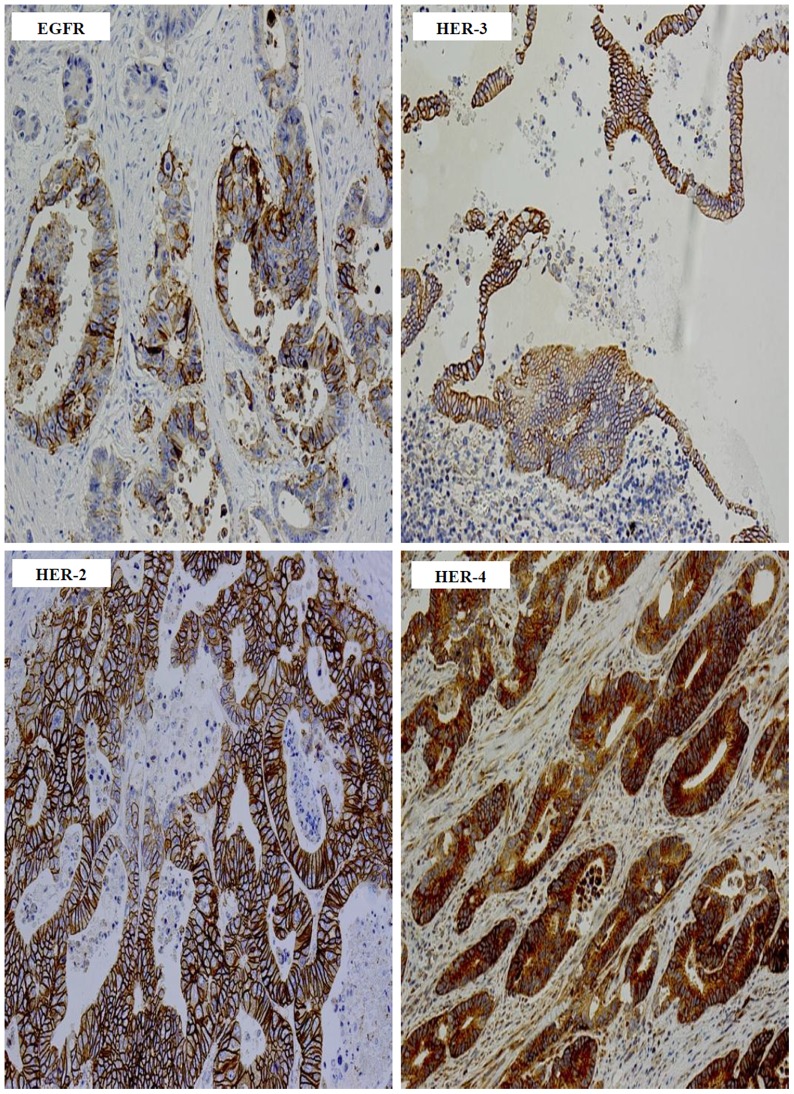
The immunostaining of HER family members in Dukes’ C and D colon cancer specimens. Immunostaining of EGFR 3+, HER-2 3+, HER-3 2+, and HER-4 3+ in formalin fixed paraffin embedded tumour sections stained immunohistochemically, as described under methods and patients section. Magnification: ×200.

**Figure 2:**
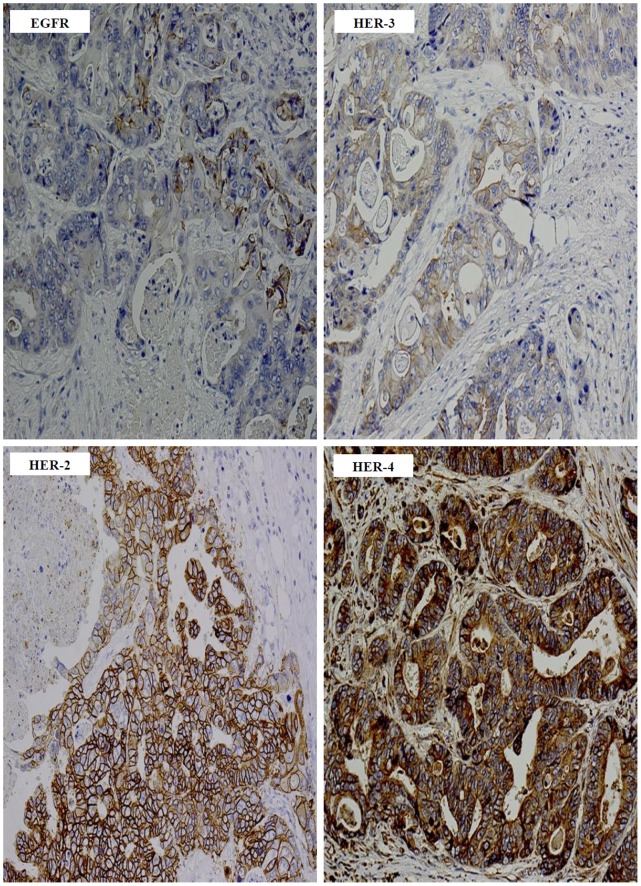
The co-expression of all HER family members in a patient with Dukes’ C colon cancer. Co-expression of EGFR, HER-2, HER-3, and HER-4 in a formalin fixed paraffin embedded tumour sections stained immunohistochemically as described under methods and patients section. Magnification: ×200.

At cut off value of ≥5%, tumour specimens from 43%, 77%, 52% and 92% of cases were EGFR, HER-2, HER-3 and HER-4 positive respectively (Table 2). In contrast to the EGFR, which had predominantly membranous staining, the predominant location of HER-2, HER-3 and HER-4 immunostaining was cytoplasmic (Table 2 & Figure 1). In this study, we have investigated the co-expression of all the members of the HER family in colon cancer patients and the results are presented in Table 3. Co-expression of EGFR with HER-2, HER-3, HER-4, and HER-2/HER-4 were present in 35%, 24%, 43% and 76% of the cases examined (Table 3). Interestingly, 18% of the patients in this study were found to co-express all four members of the HER family (Table 3 & Figure 2).

**Table 2:** Immunohistochemical expression of HER family members in Dukes’ C and D colon cancer patients.

Scoring criteria		No. of positive tumours (%)			
		EGFR	HER-2	HER-3	HER-4
**Percentage of positive tumour cells**	**>5**	37 (43)	66 (77)	45 (52)	79 (92)
	**>10**	26 (30)	10 (12)	39 (45)	79
	**>50**	3 (4)	7 (8)	19 (22)	79
**Intensity**	**1+**	30	23	31	36
	**2+**	6	41	14	45
	**3+**	1	3	0	13
**Sub-cellular localisation**	**Membranous**	30	5	21	24
	**Cytoplasmic**	7	62	39	71
	**Luminal**	0	0	31	0

**Table 3:** Co-expression level of HER family members presented as percentage positive tumour cells immunostaining in Dukes’ C and D colon cancer patients.

Markers	Number of positive tumours (%)		
	>5% cut off	>10% cut off	>50% cut off
**EGFR/HER-2**	29 (35)	5 (6)	3 (4)
**EGFR/HER-3**	20 (24)	15 (18)	3 (4)
**EGFR/HER-4**	36 (43)	25 (30)	3 (4)
**HER-2/HER-3**	38 (46)	5 (6)	1 (1)
**HER-2/HER-4**	63 (76)	10 (12)	7 (8)
**HER-3/HER-4**	43 (52)	38 (46)	18 (22)
**EGFR/HER-2/HER-3**	16 (19)	2 (2)	3 (4)
**EGFR/HER-2/HER-4**	28 (34)	5 (6)	3 (4)
**EGFR/HER-3/HER-4**	19 (23)	16 (19)	3 (4)
**HER-2/HER-3/HER-4**	36 (43)	5 (6)	1 (1)
**EGFR/HER-2/HER-3/HER-4**	15 (18)	2 (2)	0 (0)

The percentage of HER positive colon cancer cases was also evaluated when immunostaining was present in more than10% and 50% of tumour cells (Table 2). Using the cut-off values of above 10% and 50%, the percentage of cases with EGFR positive tumours were 30% and 4%, HER-2 positive tumours were12% and 8% and HER-3 positive tumours were 45% and 22%, respectively (Table 2). The number of HER-4 positive tumours remained the same regardless of the cut-off values of the percentage of positive tumour cells, as all patients had HER-4 immunostaining in more than 50% of the tumour cells in this study (Table 2).

### Expression and Co-expression of HER Family Members is Associated with Clinicopathological Parameters

The association between clinicopathological characteristics and the expression of HER family members was determined by the Chi-squared test. All sub-categories (i.e. percentage positive cells, intensity and location of the immunostaining), which were found to have a statistically significant association with clinicopathological parameters are summarised in Table 4.

**Table 4:** The association between HER family expression and clinicopathological characteristics using the Chi-squared test (Fisher’s exact test, FET).

Receptors (sub-categories)	Number of patients with receptor expression		
	Clinicopathological parameters		*P-value (FET)*
	**Age**		
	≤70	>70	
**EGFR (>5%)**	6	31	*0.022 (0.029)*
**EGFR/HER-4 (>5%)**	6	30	*0.031 (0.05)*
	**Tumour Site**		
	Right colon	Left colon	
**EGFR (>5%)**	14	23	*0.022 (0.028)*
**EGFR/HER-2 (>5%)**	10	19	*0.021 (0.024)*
**EGFR/HER-4 (>5%)**	14	22	*0.039 (0.048)*
**EGFR/HER-2/HER-4 (>5%)**	10	18	*0.036 (0.041)*
	**Dukes’ Stage**		
	C	D	
**HER-4 (>5%)**	63	16	*0.011 (0.036)*
	**T-stage**		
	T4	<T4	
**HER-3 (>10%)**	14	25	*0.037 (0.045)*
**EGFR/HER-3 (>10%)**	7	8	*0.035 (0.05)*
**HER-3/HER-4 (>10%)**	14	24	*0.026 (0.042)*
	**Grade**		
	G3	<G3	
**HER-4 (1+ intensity)**	27	9	*0.010 (0.013)*
**HER-4 (2+ intensity)**	24	21	*0.013 (0.015)*

P-value of *≤0.05* was considered significant.

When immunostaining was present in greater than 5% of tumour cells, a significant association was found between the expression of EGFR and age (*P = 0.022*), and tumour site (*P = 0.022*), with a significantly higher number of patients over 70 years having EGFR positive tumours in the left colon (Table 4). In addition, a significantly higher number of Dukes’ C tumours were found to express HER-4 (*P = 0.011*) (Table 4). Like EGFR expression, the co-expression of EGFR/HER-4 was also significantly associated with patients over 70 years old (*P = 0.031*) and presenting tumours in the left colon (*P = 0.039*) in this study. In addition, a significantly higher number of tumours in the left colon were found to co-express EGFR/HER-2/HER-4 (*P = 0.036*) (Table 4).

At the cut off value of above 10%, the expression of HER-2 was associated with involvement of more than 3 positive lymph nodes (*P = 0.047*) (Table 4). In addition, a significantly higher number of tumours expressing HER-3 (*P = 0.037*), or co-expressing EGFR/HER-3 (*P = 0.035*), or HER-3/HER-4 (*P = 0.026*) were found in tumours <pT4 stage in this study (Table 4). When analysed based on the intensity of HER staining, a significant association was found between HER-4 immunostaining intensity of 1+ and 2+ and a higher number of G3 tumours (Table 4).

### Disease-free Survival is Significantly Associated with the Expression and Co-expression of HER Family Members

**Figure 3:**
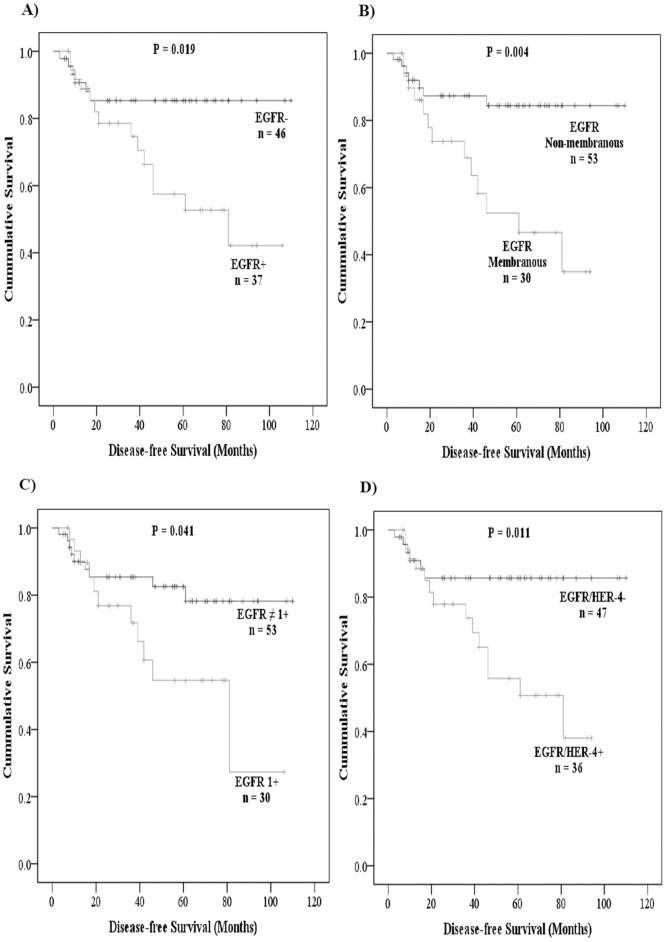
The association between EGFR and EGFR/HER-4 and disease-free survival in Dukes’ C and D colon cancer patients. Kaplan-Meier survival curves showing the impact on the disease-free survival of the patients with EGFR expression (A), membranous EGFR expression (B), EGFR 1+ immunostaining (C) and EGFR/HER-4 co-expression (D). A log-rank test value of *P≤0.05* was considered statistical significance.

The association between the expression of individual, two, three, or all four members of the HER family and disease-free survival was investigated using Kaplan-Meier curves and log rank-test. Disease-free survival was found to be significantly poorer in patients with EGFR expression at cut off values of both above 5% (*P = 0.019*) and 50% (*P = 0.005*), membranous expression of the EGFR (P = 0.004) and EGFR immunostaining intensity of 1+ (*P = 0.041*) (Figure 3 & Table 5). In addition, there was a significant association between the co-expression of EGFR and HER-4 at above 5% or above 10% of tumour cells and poorer disease-free survival (*P = 0.019*) (Table 5). The co-expression of HER-2/HER-3 was also found to be significantly associated with poorer disease-free survival (*P = 0.031*) (Table 5).

**Table 5:** The association between the expression of the HER family members and disease-free survival in univariate and multivariate analysis.

Characteristics	Univariate	Multivariate		
	*P-value*	Hazard Ratio	95% CI	*P-value*
**EGFR >5%**	*0.019*	2.96	1.13–7.71	*0.027*
**EGFR >50%**	*0.005*	6.51	1.44–29.43	*0.015*
**EGFR Membranous**	*0.004*	3.59	1.43–9.05	*0.007*
**EGFR 1+ intensity**	*0.041*	2.45	1.00–5.96	*0.048*
**EGFR/HER-4>5%**	*0.011*	3.24	1.24–8.46	*0.016*
**EGFR/HER-4>10%**	*0.044*	2.40	0.99–5.78	*0.051*
**HER-2/HER-3>50%**	*0.031*	6.93	0.89–54.23	*0.065*

P-value of *≤0.05* was considered significant.

Finally, in multivariate analysis, the expression of EGFR at cut off values of both above5% (*P = 0.027*) and 50% (*P = 0.015*), the membranous expression of EGFR (*P = 0.007*) and EGFR 1+ immunostaining (*P = 0.048*) were found to remain independent prognostic factors for poor disease-free survival. Looking at the co-expression of receptors, only EGFR/HER-4 immunostaining at above 5% tumour cells remained as an independent prognostic factor for poor disease-free survival in this study (Table 5). No significant association was found between HER expression and overall survival in this study.

## Discussion

The aberrant expression of the HER family members and, in particular, EGFR has been reported in a wide range of human cancers and has been associated with metastasis and poor prognosis [6], [25], [26]. As a result, there has been a substantial development of molecular therapies targeting EGFR and subsequent approval of anti-EGFR mAbs, such as cetuximab and panitumumab, for the treatment of patients with metastatic colorectal cancer [2], [27]. However, despite extensive studies, there are currently no reliable markers for predicting the response to therapy with these EGFR inhibitors [6], [28]. In several studies, *KRAS* mutations in patients with colorectal cancer are associated with resistance to therapy with anti-EGFR antibodies and it is the only biomarker for predicting patient outcome when treated with targeted anti-HER therapies in colorectal cancer [29]. However, despite these advancements, it is clear that not all colorectal cancer patients with wild-type KRAS would gain benefit from anti-EGFR mAbs and objective responses have also been reported in patients with KRAS mutated tumours [30]. One reason for this could be that KRAS has no direct interaction with antibody binding at the antigen site and therefore it is prudent to investigate the expression of other members of the HER family, which have been shown to activate the downstream pathways, via heterodimerisation and cross-talk, and drive the tumourigenesis of colorectal cancer in these patients [17]–[19].

Several other studies have investigated the expression of the individual members of the HER family as a prognostic indicator, yet no clear associations have been found between the expression of HER proteins and prognosis in colorectal cancer patients [31]–[37]. The reported expression of EGFR ranges from 8 to 100%, HER-2 from 1% to 89%, HER-3 from 16 to 89%, and HER-4 from 11 to 81% in colorectal cancer patients [6], [14]–[16], [20]–[22], [38]. The wide variation in the reported expression of the HER family proteins by immunohistochemistry in colorectal cancer may have contributed to the conflicting data on their prognostic significance in colorectal cancer [6]. Indeed, factors such as the use of different antibodies, differences in antigen retrieval techniques, scoring systems, different patient populations, sample sizes [e.g. tissue microarrays (TMA)], and different sample numbers could contribute to the wide variation in the reported expression of the HER family members in the literature [6], [28].

In this study, we investigated the expression of all the members of the HER family in 86 patients with Dukes’ C and D colon cancer. Since inconsistencies in the scoring system such as the usage of different cut off values for HER immunostaining is a major contributing factor for the wide variation in their reported expression in the literature, in this study immunostaining were scored and evaluated using three different cut off values of >5%, >10%, and >50% (Table 2). Of the 86 cases examined, we found 43%, 77%, 52% and 92% of the cases to have EGFR, HER-2, HER-3, and HER-4 immunostaining present in >5% of tumour cells respectively (Figure 1). Herein, while we did not find any associations between the expression of the HER family members and overall survival, this is in concordance with other studies in the literature [6]. Unlike overall survival, in this study the expression of the EGFR and co-expression of EGFR/HER-4 were associated with disease-free survival in both univariate and multivariate analysis (Figure 3 & Table 5). The expression of the EGFR has been significantly associated with poor disease-free survival and disease relapse in two other studies. In one study, Galizia et al. [34] investigated the expression of EGFR in tumour specimens from 154 Dukes’ A-D colorectal cancer patients and found membranous expression of the EGFR to be significantly associated with poor disease-free survival, both in univariate and multivariate analysis. In another study involving 102 advanced colorectal cancer patients, 75.5% of the cases were found to express EGFR, which was significantly associated with disease relapse [39]. In contrast, Leung et al. [23] in their study of 127 colon cancer patients using TMA sections, did not find any significant association between the expression of EGFR, and patient outcome, but found HER-3 expression to be a significant predictor of survival outcome. As explained above, differences such as the use of TMA sections instead of whole tissue blocks, the heterogeneous nature of tumours, and the use of different cut off values could be some of the contributing factors for the conflicting data on the expression and prognostic significance of HER proteins in colorectal cancer. In addition, as we investigated the prognostic significance of the HER family members in only 86 patients in this study, this would require further validation in larger group of colorectal cancer patients.

Several studies suggest that the heterodimerisation of HER family members plays a crucial role in tumourigenesis, and in particular they may also play an important role in the development of resistance to therapy in patients with colorectal cancer [17]–[19]. However, to date only 4 studies have determined the expression of all the individual members of the HER family, but the co-expression levels of all members of the HER family has not been previously reported in colorectal cancer [20]–[23]. To our knowledge, this is the first study to report both the individual expression and co-expression of all HER family members in tumour specimens from patients with Dukes’ C and D colon cancer. Interestingly, considering the cut-off values of 5% and 10% positive tumour cells, we found the co-expression of EGFR/HER-4 to be significantly associated with disease-free survival in patients with Dukes’ C and D colon cancer in this study (Table 5). While some studies report the associations between the co-expression of HER-2/HER-4 or HER-3/HER-4 and late tumour stages [21], [22], to our knowledge the significant co-expression of EGFR/HER-4 with poorer disease-free survival in colon cancer patients has not been previously reported. Our results suggest that heterodimer formation of EGFR and HER-4 may play an important role in the tumourigenesis of colon cancer and contribute to faster disease relapse in these patients. Interestingly, we found the co-expression of all four members of the HER family in 18% of the cases examined and 35%, 24% and 43% of the cases had co-expression of EGFR with HER-2, HER-3 and HER-4 respectively. Consequently, it is essential to investigate whether the co-expression of other members of the HER family in the EGFR positive cancers may contribute to resistance, or a poor response to therapy with the anti-EGFR mAbs cetuximab and panitumumab in patients with colorectal cancer [6].

In conclusion, the co-expression of all members of the HER family in a considerable percentage of patients with metastatic colon cancer patients reported here together with the importance of heterodimerisation between members of the HER family in the activation of HER signalling pathways, supports the need for further studies on their co-expression, prognostic significance and predictive value for response to therapy with HER inhibitors, in a larger population of colorectal cancer patients. In particular, such studies should unravel whether this sub-population of patients may benefit from therapy with the new generation of pan-HER inhibitors [40], [41].

## References

[1] Siegel R, Naishadham D, Jemal A (2013) Cancer statistics, 2013. CA Cancer J Clin 63: 11–30. 23335087 10.3322/caac.21166

[2] Wong SF (2005) Cetuximab: an epidermal growth factor receptor monoclonal antibody for the treatment of colorectal cancer. Clinical Therapeutics 27: 684–694. 16117976 10.1016/j.clinthera.2005.06.003

[3] Shih T, Lindley C (2006) Bevacizumab: an angiogenesis inhibitor for the treatment of solid malignancies. Clinical Therapeutics 28: 1779–1802. 17212999 10.1016/j.clinthera.2006.11.015

[4] Wu M, Rivkin A, Pham T (2008) Panitumumab:Human monoclonal antibody against epidermal growth factor receptor for the treatment of metastatic colorectal cancer. Clinical Therapeutics 30: 14–29. 18343240 10.1016/j.clinthera.2008.01.014

[5] Chu E (2012) An update on the current and emerging targeted agents in metastatic colorectal cancer. Clinical Colorectal Cancer 11: 1–13. 21752724 10.1016/j.clcc.2011.05.005

[6] Khelwatty SA, Essapen S, Seddon AM, Modjtahedi H (2013) Prognostic significance and targeting of HER family in colorectal cancer. Front Biosci 18: 394–421. 10.2741/4110 23276932

[7] Hebbar M, Wacrenier A, Desauw C, Romano O, Cattan S, et al (2006) Lack of usefulness of epidermal growth factor receptor expression determination for cetuximab therapy in patients with colorectal cancer. Anti-Cancer Drugs 17: 855–857. 16926635 10.1097/01.cad.0000217425.44584.9f

[8] Hecht JR, Mitchell E, Neubauer MA, Burris Iii HA, Swanson P, et al (2010) Lack of correlation between epidermal growth factor receptor status and response to panitumumab monotherapy in metastatic colorectal cancer. Clinical Cancer Research 16: 2205–2213. 20332321 10.1158/1078-0432.CCR-09-2017

[9] Siena S, Sartore-Bianchi A, Di Nicolantonio F, Balfour J, Bardelli A (2009) Biomarkers predicting clinical outcome of epidermal growth factor receptor-targeted therapy in metastatic colorectal cancer. Journal of the National Cancer Institute 101: 1308–1324. 19738166 10.1093/jnci/djp280 PMC2758310

[10] Modjtahedi H, Ali S, Essapen S (2012) Therapeutic application of monoclonal antibodies in cancer: advances and challenges. Br Med Bull 104: 41–59. 23118261 10.1093/bmb/lds032

[11] Konigsberg R, Hulla W, Klimpfinger M, Reiner-Concin A, Steininger T, et al (2011) Clinical and economic aspects of KRAS mutational status as predictor for epidermal growth factor receptor inhibitor therapy in metastatic colorectal cancer patients. Oncology 81: 359–364. 22248908 10.1159/000334919

[12] Nicholson RI, Gee JW, Harper ME (2001) EGFR and cancer prognosis. Eur J Cancer 37: 9–15. 11597399 10.1016/s0959-8049(01)00231-3

[13] McIntyre E, Blackburn E, Brown PJ, Johnson CG, Gullick WJ (2010) The complete family of epidermal growth factor receptors and their ligands are co-ordinately expressed in breast cancer. Breast Cancer Res Treat 122: 105–110. 19760033 10.1007/s10549-009-0536-5

[14] Ooi A, Takehana T, Li X, Suzuki S, Kunitomo K, et al (2004) Protein overexpression and gene amplification of HER-2 and EGFR in colorectal cancers: an immunohistochemical and fluorescent in situ hybridization study. Mod Pathol 17: 895–904. 15143334 10.1038/modpathol.3800137

[15] Cunningham MP, Essapen S, Thomas H, Green M, Lovell DP, et al (2006) Coexpression of the IGF-IR, EGFR and HER-2 is common in colorectal cancer patients. International Journal of Oncology 28: 329–335. 16391786

[16] Wei Q, Shui Y, Zheng S, Wester K, Nordgren H, et al (2011) EGFR, HER2 and HER3 expression in primary colorectal carcinomas and corresponding metastases: Implications for targeted radionuclide therapy. Oncol Rep 25: 3–11. 21109951

[17] Graus-Porta D, Beerli RR, Daly JM, Hynes NE (1997) ErbB-2, the preferred heterodimerization partner of all ErbB receptors, is a mediator of lateral signaling. EMBO J 16: 1647–1655. 9130710 10.1093/emboj/16.7.1647 PMC1169769

[18] Arteaga CL (2002) Epidermal Growth Factor Receptor Dependence in Human Tumors: More Than Just Expression? The Oncologist 7: 31–39. 12202786 10.1634/theoncologist.7-suppl_4-31

[19] Normanno N, Bianco C, De Luca A, Maiello M, Salomon D (2003) Target-based agents against ErbB receptors and their ligands: a novel approach to cancer treatment. Endocr Relat Cancer 10: 1–21. 12653668 10.1677/erc.0.0100001

[20] Baiocchi G, Lopes A, Coudry R, Rossi B, Soares F, et al (2009) ErbB family immunohistochemical expression in colorectal cancer patients with higher risk of recurrence after radical surgery. International Journal of Colorectal Disease 24: 1059–1068. 19390858 10.1007/s00384-009-0702-6

[21] Lee JC, Wang ST, Chow NH, Yang HB (2002) Investigation of the prognostic value of coexpressed erbB family members for the survival of colorectal cancer patients after curative surgery. European Journal of Cancer 38: 1065–1071. 12008194 10.1016/s0959-8049(02)00004-7

[22] Ljuslinder I, Malmer B, Isaksson-Mettävainio M, Öberg Å, Henriksson R, et al (2009) ErbB 1–4 expression alterations in primary colorectal cancers and their corresponding metastases. Anticancer Research 29: 1489–1494. 19443355

[23] Leung SP, Griffith OL, Masoudi H, Gown A, Jones S, et al (2008) Clinical utility of type 1 growth factor receptor expression in colon cancer. Am J Surg 195: 604–610. 18424279 10.1016/j.amjsurg.2007.12.032

[24] Cunningham MP, Thomas H, Fan Z, Modjtahedi H (2006) Responses of Human Colorectal Tumor Cells to Treatment with the Anti–Epidermal Growth Factor Receptor Monoclonal Antibody ICR62 Used Alone and in Combination with the EGFR Tyrosine Kinase Inhibitor Gefitinib. Cancer Research 66: 7708–7715. 16885373 10.1158/0008-5472.CAN-06-1000

[25] Ciardiello F, Tortora G (2008) EGFR Antagonists in Cancer Treatment. New England Journal of Medicine 358: 1160–1174. 18337605 10.1056/NEJMra0707704

[26] Mendelsohn J, Baselga J (2003) Status of epidermal growth factor receptor antagonists in the biology and treatment of cancer. Journal of Clinical Oncology 21: 2787–2799. 12860957 10.1200/JCO.2003.01.504

[27] Giusti RM, Shastri K, Pilaro AM, Fuchs C, Cordoba-Rodriguez R, et al (2008) U.S. Food and Drug Administration Approval: Panitumumab for Epidermal Growth Factor Receptor–Expressing Metastatic Colorectal Carcinoma with Progression Following Fluoropyrimidine-, Oxaliplatin-, and Irinotecan-Containing Chemotherapy Regimens. Clinical Cancer Research 14: 1296–1302. 18316547 10.1158/1078-0432.CCR-07-1354

[28] Modjtahedi H, Essapen S (2009) Epidermal growth factor receptor inhibitors in cancer treatment: advances, challenges and opportunities. Anti-Cancer Drugs 20: 851–855 810.1097/CAD.1090b1013e3283330590. 10.1097/CAD.0b013e3283330590 19826350

[29] Dempke WC, Heinemann V (2010) Ras mutational status is a biomarker for resistance to EGFR inhibitors in colorectal carcinoma. Anticancer Res 30: 4673–4677. 21115922

[30] Stintzing S, Fischer von Weikersthal L, Decker T, Vehling-Kaiser U, Jager E, et al (2012) FOLFIRI plus cetuximab versus FOLFIRI plus bevacizumab as first-line treatment for patients with metastatic colorectal cancer-subgroup analysis of patients with KRAS: mutated tumours in the randomised German AIO study KRK-0306. Ann Oncol 23: 1693–1699. 22219013 10.1093/annonc/mdr571

[31] Kluftinger AM, Robinson BW, Quenville NF, Finley RJ, Davis NL (1992) Correlation of epidermal growth factor receptor and c-erbB2 oncogene product to known prognostic indicators of colorectal cancer. Surg Oncol 1: 97–105. 1285215 10.1016/0960-7404(92)90062-p

[32] Spano J-P, Lagorce C, Atlan D, Milano G, Domont J, et al (2005) Impact of EGFR expression on colorectal cancer patient prognosis and survival. Ann Oncol 16: 102–108. 15598946 10.1093/annonc/mdi006

[33] Theodorpoulos GE, Karafoka E, Papailiou JG, Stamopoulos P, Zambirinis CP, et al (2009) p53 and EGFR Expression in Colorectal Cancer: A Reappraisal of ‘Old’ Tissue Markers in Patients with Long Follow-up. Anticancer Research 29: 785–791. 19331236

[34] Galizia G, Lieto E, Ferraraccio F, De Vita F, Castellano P, et al (2006) Prognostic Significance of Epidermal Growth Factor Receptor Expression in Colon Cancer Patients Undergoing Curative Surgery. Annals of Surgical Oncology 13: 823–835. 16614884 10.1245/ASO.2006.05.052

[35] Takemura K, Obara T, Okano S, Yokota K, Ura H, et al (1991) Immunohistochemical study on the expression of epidermal growth factor receptor (EGFR) in human colorectal carcinomas. Nihon Shokakibyo Gakkai Zasshi 88: 1177–1183. 1880949

[36] Scartozzi M, Bearzi I, Berardi R, Mandolesi A, Fabris G, et al (2004) Epidermal growth factor receptor (EGFR) status in primary colorectal tumors does not correlate with EGFR expression in related metastatic sites: Implications for treatment with EGFR-Targeted Monocolonal Antibodies. Journal of clinical oncology 22: 4772–4778. 15570078 10.1200/JCO.2004.00.117

[37] Scartozzi M, Mandolesi A, Giampieri R, Bittoni A, Pierantoni C, et al (2011) The Role of HER-3 Expression in the Prediction of Clinical Outcome for Advanced Colorectal Cancer Patients Receiving Irinotecan and Cetuximab. Oncologist 16: 53–60. 21212430 10.1634/theoncologist.2010-0119 PMC3228051

[38] Maurer CA, Friess H, Kretschmann B, Zimmermann A, Stauffer A, et al (1998) Increased expression of erbB3 in colorectal cancer is associated with concomitant increase in the level of erbB2. Human Pathology 29: 771–777. 9712416 10.1016/s0046-8177(98)90444-0

[39] Goldstein NS, Armin M (2001) Epidermal growth factor receptor immunohistochemical reactivity in patients with American Joint Committee on Cancer Stage IV colon adenocarcinoma: implications for a standardized scoring system. Cancer 92: 1331–1346. 11571750 10.1002/1097-0142(20010901)92:5<1331::aid-cncr1455>3.0.co;2-m

[40] Khelwatty SA, Essapen S, Seddon AM, Modjtahedi H (2011) Growth response of human colorectal tumour cell lines to treatment with afatinib (BIBW2992), an irreversible erbB family blocker, and its association with expression of HER family members. Int J Oncol 39: 483–491. 21617858 10.3892/ijo.2011.1054

[41] Britten CD (2004) Targeting ErbB receptor signaling: a pan-ErbB approach to cancer. Mol Cancer Ther 3: 1335–1342. 15486201

